# Licury Cake in Diets for Lactating Goats: Intake, Digestibility, Feeding Behavior, Milk Production and Composition, and Nitrogen Metabolism

**DOI:** 10.3390/ani13152535

**Published:** 2023-08-06

**Authors:** Fernanda G. Ferreira, Laudí C. Leite, Henry D. R. Alba, Douglas dos S. Pina, Stefanie A. Santos, Manuela S. L. Tosto, Carlindo S. Rodrigues, Robério R. Silva, José E. de Freitas Júnior, Bruna M. A. de C. Mesquita, Gleidson G. P. de Carvalho

**Affiliations:** 1 Department of Animal Science, Universidade Federal da Bahia, Av. Adhemar de Barros, 500, Ondina, Salvador 40170110, Brazil; fernandagazarufrb@gmail.com (F.G.F.); henry.ruiz@ufba.br (H.D.R.A.); douglas.pina@ufba.br (D.d.S.P.); stefanie.alvarenga@ufba.br (S.A.S.); mtosto@ufba.br (M.S.L.T.); carlindo.rodrigues@ufba.br (C.S.R.); jose.esler@ufba.br (J.E.d.F.J.); 2 Department of Animal Science, Universidade Federal do Recôncavo da Bahia, Cruz das Almas 44380000, Brazil; laudi.ufrb@gmail.com; 3 Department of Animal Science, Universidade Federal do Sudoeste da Bahia, Itapetinga 45700000, Brazil; rrsilva.uesb@hotmail.com; 4 Institute of Agricultural Sciences, Universidade Federal de Minas Gerais, Montes Claros 39404547, Brazil; brunacarvalho@ufmg.br

**Keywords:** by-product, metabolism, performance, ruminant nutrition, small ruminant

## Abstract

**Simple Summary:**

Licury cake is a by-product used to replace conventional ingredients in ruminant diets to reduce production costs. Some studies in sheep and in beef and dairy cattle showed promising results with the inclusion of licury cake in the diets of feedlot ruminants. The main objective of this study was to evaluate the productive performance and metabolic behavior of dairy goats subjected to increasing inclusion levels of licury cake in the diet. Our main results showed that the inclusion of licury cake in the diet did not promote negative effects on the nutrient intake, digestibility, metabolism, and performance of feedlot lactating goats. We additionally observed that, through feeding behavior, the inclusion of licury cake promoted greater efficiency in the intake and rumination of fiber. Moreover, through the metabolism, we found that the inclusion of licury cake promoted higher recycling of nitrogen; however, more studies are needed to elucidate these facts. Our results showed that the inclusion of licury cake of up to 200 g kg^−1^ of dry matter in the diets of lactating goats can be recommended without affecting productive performance.

**Abstract:**

The objective of this study was to determine the effects of licury cake (LC) inclusion in the diets of lactating goats on productive and metabolic performance. Twelve lactating goats, eight Saanen and four Anglo-Nubian, were distributed in a triplicate 4 × 4 Latin square design, with four treatments (0, 66.7, 133.3, and 200 g kg^−1^ of dry matter—DM). On the one hand, the LC inclusion increased neutral detergent fiber, indigestible neutral detergent fiber, and potentially digestible neutral detergent fiber (*p* < 0.001) intake. On the other hand, LC inclusion reduced ether extract and non-fibrous carbohydrate (*p* < 0.001) intake. There was a reduction in dry matter digestibility (*p* = 0.018) and an increase in neutral detergent fiber digestibility (*p* = 0.036). Feeding (*p* = 0.005) and rumination (*p* < 0.001) efficiencies increased with LC inclusion. The nitrogen balance was similar for all tested diets; however, we observed recycling metabolism. Based on the studied parameters, mainly milk production and composition, we recommend the LC inclusion of up to 200 g kg^−1^ DM in diets for lactating goats.

## 1. Introduction

The use of intensive systems (feedlots) for the production of dairy animals is considered an important strategy to control the proper management of animals, especially the nutritional management, which can greatly affect the productive performance of the animals [1]. This type of system ensures the continuity of protein products of animal origin to satisfy the demand for human food, both in quantity and quality.

However, intensive production systems are usually associated with higher costs. Animal feed represents the highest proportion of production costs, accounting for nearly 70% of the total cost [2]. The use of alternative feed ingredients, which can replace conventional and more expensive ones (such as corn ground and soybean meal), is an important strategy to reduce production costs and, consequently, improve the economic performance of the activity.

Several agro-industrial by-products have been suggested in the literature as alternative feeds for animal nutrition that can contribute to cost reductions. The licury tree (*Syagrus coronata*) is a palm species highly resistant to drought in semi-arid regions. The fruit is composed of a fibrous pericarp and an oil-rich endosperm (almonds). In Brazil, licury oil is the suitable for the manufacture of detergents, powdered soap, and bar soap. Furthermore, licury products are successfully used in human nutrition in the form of *in natura*, cereal bars, yogurt, and licury flour, among others [3]. From the process of extracting the oil from the almonds by the pressing method, between 30 and 40% of licury cake (LC) is obtained [4]. Ferreira et al. [5] reported that the inclusion of LC in the feed of lactating cows reduced the cost of concentrates to 38.5%, which shows its potential in animal feed and a considerable reduction in production costs.

In addition to having lower commercial value compared to ingredients that are traditionally used in animal feed, LC has an additional favorable composition: an average of 221.8 g of crude protein (CP) kg^−1^ dry matter (DM), 60.7 g of ether extract (EE) kg^−1^ DM, and 486.0 g of neutral detergent fiber (NDF) kg^−1^ DM [6].

Various studies have already evaluated the use of by-products in animal feed but studies with licury cake in lactating goat feeding are scarce. The results obtained with the inclusion of LC in the feeding of ruminants are different among the species and the categories, resulting in controversial conclusions about the adequate amount of LC addition in the diets of ruminants. In feedlot systems for beef cattle, the use of LC in the diet at a level of 85.0 g kg^−1^ DM is recommended [7]. For feedlot meat goats, in highly concentrated diets, the inclusion of LC of up to 100 g kg^−1^ DM is accepted [5]. In grazing lambs supplemented with LC, the inclusion of 174 g kg^−1^ DM of the total diet was found to be the ideal level of inclusion [8]. In grazing lactating cows, the ideal LC inclusion level was observed to be up to 70 g kg^−1^ DM based on the total diet [9].

Given the results obtained from studies that have analyzed the inclusion of LC in the ruminant diet, we can assume that this by-product can be included in diets and promote higher productive performance in feedlot dairy goats without affecting animal metabolism and product quality. In this context, the objective of the current study was to evaluate the inclusion of increasing levels of LC (0, 66.7, 133.3, and 200 g kg^−1^ DM) in the diets of feedlot lactating goats on the intake and digestibility of nutritional components, feeding behavior, productive performance, milk composition, blood metabolites, and nitrogen balance.

## 2. Materials and Methods

### 2.1. Ethics Committee and Experiment Location

The use of goats in this study was approved by the Ethics Committee for the Use of Animals (CEUA) of the Federal University of Bahia (UFBA) under registration number 73/2018.

The experiment was conducted from October to December 2018, in the goat sector of the UFBA Experimental Farm, located in the Entre Rios municipality, Bahia, Brazil (11°56′31″ S, 38°05′04″ W, altitude 162 m). The region has an average minimum temperature of 22 °C, an average maximum temperature of 29 °C, average annual rainfall of 1000 to 1251 mm, and a warm semi-humid climate.

### 2.2. Animals, Experimental Design, and Management

Eight Saanen and four Anglo-Nubian goats, multiparous, with an average body weight of 37.93 ± 9.22 kg (mean ± standard deviation), an average of 30 days of lactation, and an average production of 0.7 kg of milk day^−1^ were used. Goats were distributed in a Latin square design (4 × 4) in triplicate, considering four periods and four animals by square. One square was created with Anglo-Nubian goats, and Saanen goats were randomly distributed in the other two squares. 

Each experimental period lasted fourteen days, with the last four days dedicated to sample and data collection. The diets were formulated according to the NRC [10] to meet the maintenance and milk production requirements of lactating goats. Treatments consisted of a diet without the inclusion of LC and three with the inclusion of LC at levels of 66.7, 133.3, and 200 g kg^−1^ DM of the total diet (Table 1). In order to keep the diets as isonitrogenous and isoenergetic as possible, we partially replaced ground corn and cottonseed meal as LC was included in the diet. The forage used was corn silage and the forage:concentrate ratio was 50:50.

The animals were managed in individual pens of 1.5 m^2^, with a suspended floor and wooden slats, equipped with feeders and drinkers with free access to water and feed.

Diets were provided as a total mixed ration, twice daily (08:00 and 15:00). The supplied diet and leftovers were weighed daily to control the daily intake of nutritional compounds of the animals, and to adjust the supplied feed to guarantee approximately 20% leftovers.

Animals were milked daily, manually, once a day (07:00), using 0.5% iodized glycerin for pre- and post-milking. The process was carried out in previously sanitized boxes, following the regulatory hygiene practices for milking. The first streams of milk were poured into a glass with a dark bottom to detect possible cases of mastitis.

### 2.3. Intake and Apparent Digestibility of Nutritional Components

The intake of nutritional compounds (g day^−1^) was calculated as the difference between the amount of the nutrient present in the diet and the amount of the nutrient contained in the leftovers. The diet and leftover sample collections were carried out in the last four days of each experimental period.

The apparent digestibility was determined by the indirect method using spot feces collection. Feces were collected directly from the rectal ampulla between days 12 and 14 of each experimental period, twice daily, as follows: day 12—8:00 and 14:00, day 13—10:00 and 16:00, and day 14—12:00 and 18:00. After collection, feces were weighed and placed in a forced ventilation oven at 55 °C. The samples were then weighed and stored for further analysis. After the digestibility trial, the diet, leftover, and feces samples were ground to 1 mm for the determination of DM, ash, EE, CP, NDF, and non-fibrous carbohydrates (NFC). Total fecal excretion was estimated using non-digestible neutral detergent fiber (NDFi) as an internal indicator [11].

To estimate the apparent digestibility of the nutritional components, we used the values of the nutrient intake and the nutrient excretion in feces. The digestibility coefficients (*DC*) of DM, CP, NDF, and EE were calculated according to Berchielli et al. [12] as follows:

$$DC=\left[\frac{kg\;ingested\;fraction-kg\;excreted\;fraction}{kg\;ingested\;fraction}\right]\times 100$$

### 2.4. Feeding Behavior

The animals were subjected to visual observation (day 11 of each experimental period), during a 24-h period, with 5-min intervals, to assess feeding, rumination, and idling times [13]. Feeding behavior data were recorded by eight trained raters, which were strategically positioned so that they did not interfere with the natural behavior of the animals. During nightly evaluations, the environment was maintained with artificial light, and the goats were adapted to these conditions three days before the evaluation. Feeding behavior data were obtained according to the methodology described by Bürger et al. [14].

The time spent feeding, ruminating, and idling was obtained by multiplying the frequency of each activity by the time interval of the observations. Periods were obtained by observing the number of episodes in a 24-h interval. Feeding and rumination efficiencies of DM and NDF were calculated by dividing the intake of these nutrients by the time spent feeding and ruminating.

### 2.5. Milk Production and Composition

Daily milk production was recorded by weighing the milk throughout the experimental period; unusual days (decreased production due to stress or illness) were not observed in the analysis.

For the analysis of the chemical composition, we collected milk samples at the time of milking. The collected samples were stored in a plastic bottle with 2-bromo 2-nitropropane 1-3-diol (bromopol) preservative, for further analysis of protein, fat, lactose, urea, and total solids, using the Bentley-2000 Infrared Analyzer instrument (Bentley Instruments Inc., Curitiba, Paraná, Brazil). Between the collection time and the analysis of the samples, up to 48 h elapsed. The corrected milk fat (CMF) at 4% was obtained according to the following equation: CMF = 0.4 × milk production (g day^−1^) + 15 × milk fat (g day^−1^) [15].

Somatic cell counting (SCC) was performed using a Bentley Somacount-500 device (Bentley Instruments Inc., Curitiba, Paraná, Brazil). The somatic cell score (SCS) was estimated using the following equation: SCS = log base 2 (SCC 100,000^−1^) + 3 [16]. These analyses were carried out in the laboratory of Clínica do Leite ESALQ/USP, in Piracicaba-SP, Brazil.

### 2.6. Chemical Analysis

We processed and analyzed the ingredients, diets, leftovers, feces, and urine at the Animal Nutrition Laboratory, UFBA School of Veterinary Medicine and Animal Production. We dried all samples in a forced ventilation oven at 55 °C for 72 h. After pre-drying and grinding, we ground samples in a Willey-type mill equipped with 1-mm diameter sieves for bromatological composition analysis and 2-mm diameter sieves for NDFi evaluation. The analyses performed in 1-mm samples were DM (method 934.01), ash (method 930.06), EE (method 920.39), and CP (method 981.10) [17].

For the analysis of NDF and acid detergent fiber (ADF), we used the methodology proposed by Van Soest et al. [18]. NDF was assayed with a heat-stable amylase. NDFap and ADFap were expressed exclusively of residual ash [19] and protein [20]. Lignin was determined according to the AOAC 973.18 [17] method.

Non-digestible neutral detergent fiber (NDFi) was determined by incubating the 2-mm samples by the in situ method, using TNT bags (100 g m^2^), following the methodology described by Valente et al. [21]. Potentially digestible neutral detergent fiber (NDFpd) was obtained as the difference between NDFap and NDFi.

To estimate the value of non-fibrous carbohydrates, we used the equation suggested by Hall [22]: NFC (%) = 100 − (%CP + %EE + %ash + %NDFap).

The total digestible nutrients (TDN) were estimated using the formulas suggested by Da Cruz et al. [23] for small ruminants. The metabolizable energy was obtained from the NRC equation [15].

### 2.7. Blood Metabolites

We collected blood samples on day 14 of each experimental period, four hours after morning feeding, by venipuncture of the jugular vein in native tubes (Vacutainer). We immediately centrifugated blood samples at 3500 rpm for 15 min to obtain blood serum. Serum samples were then stored in Eppendorf tubes and stored in a freezer at −20 °C for further analysis.

We used the colorimetric method to determine the serum concentrations of albumin, total protein, and urea; we used commercial kits in our analysis (Doles Re-agentes Ltd., Goiânia, Goiás, Brazil), and readings were carried out in a spectrophotometer (AJX- 1900, Micronal SA, São Paulo, Brazil).

### 2.8. Nitrogen Balance

We collected urine spot samples on day 13 of each experimental period, approximately 4 h after feeding, during spontaneous urination. Immediately, urine aliquots of 10 mL were collected, which were diluted in 40 mL of 0.036 N sulfuric acid, as described by Valadares et al. [24]. To estimate the daily urinary excretion, the creatinine content of the samples was determined using a commercial kit (Labtest^®^, Lagoa Santa, Minas Gerais, Brazil) and reading in a spectrophotometer (AJX-1900, Micronal SA, São Paulo, Brazil). We used the formula suggested by Fonseca et al. [25], which considers average creatinine excretion of 26.05 mg kg^−1^ of body weight (BW) for lactating goats. Daily urinary excretion (L day^−1^) = (26.05 × BW, kg) (creatinine excretion, mg L^−1^)^−1^.

The nitrogen content of the urine and feces samples was determined by the Kjeldahl method [17]. The balance of nitrogen compounds was obtained using the formulas suggested by Zeoula et al. [26].

### 2.9. Statistical Analysis

We ran our statistics analysis using the statistical software SAS version 9.2 (Statistical Analysis System, 2009) [27]. The variables of intake, digestibility, feeding behavior, milk production and composition, and nitrogen metabolism were assessed according to a triplicated 4 × 4 Latin square. The mathematical model below was applied:

$${\hat{Y}}_{\mathrm{ijkl}}=\mu +{\mathrm{LS}}_{i}+A{\left({\mathrm{LS}}_{i}\right)}_{j}+P_{k}+{\mathrm{LC}}_{l}+{\mathrm{LS}}_{i}\times {\mathrm{LC}}_{l}+{Ɛ}_{\mathrm{ijkl}}$$

where Ŷ_ijkl_ = dependent variable; μ = overall mean; LS_i_ = fixed effect of the Latin square (i = 1, 2 and 3); A(LS_i_)_j_ = random effect of the animal into the Latin square (j = 1, 2, 3 and 4); P_k_ = random effect of the period (k = 1, 2, 3 and 4); LC_l_ = effect of the LC level (l = 0, 80, 160, and 240 g kg^−1^); LS_i_ × LC_l_ = fixed effect of the interaction between Latin square and LC inclusion level; and Ɛ_ijkl_ = random experimental error associated with each observation, with NID ~ (0, σ2) assumption. 

Furthermore, we evaluated the effect of the LC inclusion level using Orthogonal Polynomial Contrasts to determine the linear (−3, −1, +1, +3) and quadratic (+1, −1, −1, +1) effects. We considered the level of a 5% probability of type I error (*p* ≤ 0.05) in our study. No interaction between treatment and racial group was observed for any of the variables studied.

## 3. Results

### 3.1. Intake and Apparent Digestibility of Nutritional Components

The inclusion of LC in the diets of lactating goats did not affect the intake of DM (*p* = 0.05), CP (*p* > 0.05), and organic matter (*p* > 0.05). On the other hand, 1 g of LC kg^−1^ DM resulted in an increase of 0.72, 0.28, and 0.45 g day^−1^ in the intake of NDF (*p* < 0.001), NDFi (*p* < 0.001), and NDFpd (*p* < 0.001), respectively. There was a reduction of 0.003 and 0.690 g day^−1^ in the intake of ether extract (*p* = 0.035) and NFC (*p* < 0.001), respectively (Table 2).

Dry matter digestibility decreased linearly (*p* = 0.018) and NDF digestibility increased linearly (*p* = 0.036) due to the inclusion of LC in goat diets (Table 2). 

### 3.2. Feeding Behavior

The parameters related to feeding behavior were not influenced by the inclusion of LC in the diets, except for the NDF feed efficiency (*p* = 0.005) and the NDF rumination efficiency (*p* < 0.001), which increased proportionally to the increase in the inclusion of LC (Table 3).

### 3.3. Production and Composition of Milk

The inclusion of LC in the diets of lactating goats did not influence milk production, resulting in average production of 1017.3 g day^−1^. The composition of the milk was not influenced, with the exception of milk urea (*p* = 0.026), which linearly increased with the inclusion of LC in the diet (Table 4).

### 3.4. Blood Metabolites

There was no effect on albumin (*p* > 0.05), globulin (*p* > 0.05), the albumin:globulin ratio (*p* > 0.05), and total protein (*p* > 0.05) in the serum of goats fed increasing inclusion levels of LC. However, a quadratic effect was observed in serum urea (*p* = 0.044), with a maximum value of 81.48 mg dL^−1^ with the estimated inclusion level of 156.22 g of LC kg^−1^ DM (Table 5).

### 3.5. Nitrogen Balance

No differences were verified in the nitrogen balance parameters up to the level of inclusion of 200 g kg^−1^ of LC in the diets of lactating goats (Table 6).

## 4. Discussion

### 4.1. Intake and Apparent Digestibility of Nutritional Components

In the current study, licury cake was ground to be added to the diets of lactating goats. For this reason, despite the high NDF content in LC (64% DM), its physical form did not affect diet retention in the rumen. Therefore, it is unlikely that the fiber components in this ingredient had a negative influence on feed intake due to a possible increase in rumen distension observed with high NDF feeds that may promote satiety, justifying the lack of effect on DM intake. The reductions in EE intake and NFC intake can be attributed to the low concentrations of these nutrients in LC (33.9 and 120.2 g kg^−1^ DM, respectively). Corroborating our results, Bagaldo et al. [8] found no differences in DM intake and a reduction in NFC intake with the inclusion of LC in the diets of feedlot lambs.

According to Van Soest [18], lignin is the main limiting factor of digestibility, since it is indigestible and makes the nutritional components associated with it also indigestible. However, the nutritional components that are not associated with lignin are free of the negative effects on digestibility. In this way, the increase in the lignin content of the diet with the inclusion of LC, as well as the increase in the NDF intake, contributed to the reduction in DM digestibility.

An increase in NDF digestibility is associated with lower intake of NFC, as well as higher NDFpd intake, since this set of factors promotes ideal rumen pH values (6.2–6.8) for the development and maintenance of fibrolytic bacteria [28,29]. A negative correlation between NFC intake, in high-grain diets, and the population of fibrolytic bacteria was reported by Zhang et al. [30], in a study with goats. Therefore, the inclusion of LC probably modified the pH of the rumen environment, resulting in an ideal environment for fiber-degrading bacterial species. Consequently, as a result, we observed an increase of 0.018% in NDF digestibility for each g of LC kg^−1^ DM added to the diet.

### 4.2. Feeding Behavior

According to Van Soest [18], the times spent in feeding and rumination are influenced by the physical form of the diet. Schultz et al. [31], who evaluated different fiber diet content levels and particle sizes in lactating goats, reported that the particle size had an effect on feeding behavior. Therefore, the similarity of the forage:concentrate ratio of the diets, and the physical form of the LC (ground), influenced the results, so that the effects of the higher fiber intake from the LC did not influence feeding behavior. This was supported by the result that no differences were observed in the time spent in feeding, rumination, and idling.

A similar result was obtained by Santos et al. [7], who studied the inclusion of LC in the feeding of steers. The authors found no difference in the times spent in feeding, rumination, and idling. The increase in the feeding and rumination efficiencies of neutral detergent fiber (NDF) was justified, due to the increase in the intake of this fraction from the diet with the same feeding and rumination time.

### 4.3. Production and Composition of Milk

Milk production is positively correlated with nutrient intake [32]. Therefore, the factors that interfere with intake will directly influence the production and composition of milk. Another parameter that has a high correlation with milk production is the percentage of lactose in milk [33], which is the component of milk that has an osmotic function in the mammary gland and is the main milk volume regulator [34]. In this way, the similarity in the volume of milk produced is justified by the similarities in the DM intake and lactose content of the milk.

Milk fat comes from de novo synthesis in the mammary gland, diet, and the mobilization of body proteins [35]. An increase in NDF intake promotes higher concentrations of acetic acid from fermentation in the rumen. The production of acetic acid in the rumen is important considering that this is the main source for the “de novo synthesis” of fat in the mammary gland [36]. On the other hand, there was a reduction in EE intake, leading to lower dietary fat intake. However, neither the increase in NDF intake nor the reduction in EE intake were inefficient in changing the fat content of the milk.

The amino acids used for the synthesis of milk proteins in the mammary gland are mainly amino acids from the metabolism of rumen microbiota and dietary proteins. Therefore, the amount of substrate available for the rumen microbiota growth is reflected in a higher contribution of amino acids to the mammary gland [37]. In this way, similarity in CP intake resulted in similarity in milk protein content as LC was included in the diet. 

Corroborating the present study, Ferreira et al. [5] found no differences in the production and composition of milk from cows supplemented on pasture with concentrate containing LC up to 600 g kg^−1^ DM.

No effect was found on somatic cell count (576.8 × 1000 mL^−1^–1400.0 × 1000 mL^−1^) or score (5.53–6.81); however, high values were observed. According to Koop et al. [38], goat milk with a somatic cell count less than 1500 × 1000 mL^−1^ is acceptable to evaluate goats as physiologically healthy [39]. Infected goats with symptoms have somatic cell count levels of 2000 × 1000 mL^−1^ to 14,000.0 × 1000 mL^−1^ [40]. These higher values are normal in goat milk compared to sheep and cow milk due to the greater loss of secretory gland cells as a result of milk production [41,42].

The observed increase in milk urea is indicative of nitrogen recycling. The inclusion of licury cake in the diet was not enough to promote changes in CP intake or digestibility, although the protein content of the diet decreased. However, the lignin content and NDF intake were higher with the inclusion of LC in the diet. These fractions likely affected protein degradability and digestibility [43], resulting in low protein uptake that promoted nitrogen recycling, as occurs in low-protein diets [44]. Another explanation could be changes in rumen microbiota, mainly proteolytic bacteria and protozoa, which may increase rumen ammonia production, resulting in increased nitrogen recycling [45]. This recycling is in the form of urea, transported through the blood and reaching the mammary gland in the recycling process [46]. The recycling theory can be corroborated by the observed serum urea in blood metabolites.

### 4.4. Blood Metabolites

Total protein and serum albumin concentrations were maintained, probably due to the similarity between CP intake and CP digestibility, since their concentration is associated with the quantity and quality of dietary protein [47].

The inclusion of LC in the diet decreased the NFC intake. This could have affected the synchronization between protein and energy, since the CP intake was similar between diets, i.e., it did not follow the behavior of the NFC intake. For the microorganism to transform the nitrogen available in the rumen environment into microbial protein, it requires energy [48]. Therefore, the excess protein in the rumen, due to a lack of energy, was transformed into ammonia, absorbed by the rumen wall, and transported to the liver, where it was transformed into urea, a less toxic substance. Urea synthesized in the liver was released into the bloodstream [49], which explains the increase in serum urea.

### 4.5. Nitrogen Balance

No difference in nitrogen balance was observed in lactating goats as a function of LC inclusion, similarly reflected in CP intake and CP digestibility. In accordance with the results of the present study, Ferreira et al. [4] studied the inclusion of LC in the supplementation of grazing lactating cows and did not observe a difference in the nitrogen balance parameters evaluated. However, the quadratic trend in urinary nitrogen excretion probably correlates with the nitrogen recycling theory. In this sense, further studies on the use of LC in diets for lactating goats are recommended.

The similarity in the retained nitrogen indicates that the ingested nitrogen was sufficient to satisfy the protein needs of the animal for maintenance and milk production up to the level of LC inclusion in the diet of 200 g kg^−1^ DM. 

## 5. Conclusions

Considering the obtained results, the inclusion of licury cake in up to 200 g kg^−1^ DM is recommended for the diets of lactating goats, since it does not affect the productive parameters and the composition of the milk. This makes licury cake a positive replacement for conventional ingredients used in ruminant diets, with the objective of using local ingredients and in some cases offering low-cost ingredients.

## Figures and Tables

**Table 1:** Proportions of ingredients and chemical compositions of the experimental diets.

Variable	Licury Cake (g kg^−1^)				Licury Cake
	0	67	133	200	
	Ingredient (g kg^−1^)				
Maize silage	500.0	500.0	500.0	500.0	-
Licury cake	0.0	67.0	133.0	200.0	-
Ground corn	225.0	183.0	146.0	108.0	-
Cottonseed meal	200.0	175.0	146.0	117.0	-
Corn germ	17.0	17.0	17.0	17.0	-
Soybean meal	42.0	42.0	42.0	42.0	-
Urea	8.0	8.0	8.0	8.0	
Mineral supplement ^1^	8.0	8.0	8.0	8.0	-
	Chemical composition (g kg^−1^ DM)				
Dry matter (g/kg as-fed)	620.0	620.7	621.3	621.8	913.2
Mineral matter	34.0	33.0	30.5	35.1	32.1
Crude protein	184.6	184.2	182.8	178.0	176.6
Neutral detergent fiber ap ^2^	382.6	411.8	440.0	468.3	637.2
Acid detergent fiber ap ^2^	258.8	279.4	299.1	318.8	487.4
Potentially digestible neutral detergent fiber	223.2	240.1	256.4	272.7	368.8
Neutral detergent insoluble nitrogen	-	-	-	-	13.7
Acid detergent insoluble nitrogen	-	-	-	-	3.1
Lignin	81.5	90.2	98.7	107.1	218.2
Ether extract	40.6	39.4	38.0	36.6	33.9
Non-fibrous carbohydrates	374.5	323.3	299.6	273.0	120.2
Total digestible nutrients	768.7	735.6	725.9	710.7	610.6
Metabolizable energy, Mcal kg^−1^	2.97	2.83	2.78	2.71	2.27

^1^ Provides per kilogram of active element: calcium—183.00 g, phosphorus—60.00 g, potassium—28.00 g, sulfur—16.00 g, magnesium—20.00 g, copper—250.00 mg, cobalt—30.00 mg, chromium—10.00 mg, iron—250.00 mg, iodine—70.00 mg, manganese—1500.00 mg, selenium—30.00 mg, zinc—3500.00 mg, fluorine (max.)—600.00 mg. ^2^ Assayed with a heat-stable amylase and expressed exclusively of residual ash and protein.

**Table 2:** Intake and apparent digestibility of nutritional components in lactating goats fed diets with increasing levels of licury cake.

Variable	Licury Cake (g kg^−1^)				SEM ^1^	*p*-Value	
	0	67	133	200		Linear	Quadratic
	Nutritional component intake (g day^−1^)						
Dry matter	1596.0	1653.8	1593.1	1606.7	47.95	0.876	0.591
Organic matter	1542.8	1597.8	1539.2	1551.4	46.23	0.852	0.589
Crude protein	297.5	307.3	288.9	292.0	9.19	0.392	0.714
Ether extract ^2^	65.9	67.2	62.9	60.9	1.91	0.035	0.412
Neutral detergent fiber ^3^	588.6	654.7	684.3	739.1	22.26	<0.001	0.732
Non-digestible neutral detergent fiber ^4^	242.8	268.7	278.2	301.0	9.27	<0.001	0.826
Potentially digestible neutral detergent fiber ^5^	345.8	386.1	406.0	438.1	13.07	<0.001	0.673
Non-fibrous carbohydrates ^6^	636.1	615.0	548.6	504.4	17.88	<0.001	0.469
Total digestible nutrients	1183.2	1213.5	1176.3	1162.4	34.34	0.494	0.497
	Nutritional component digestibility (%)						
Dry matter ^7^	68.9	67.3	67.6	66.3	0.40	0.018	0.786
Crude protein	73.8	73.3	72.2	75.0	0.53	0.575	0.107
Ether extract	90.5	92.5	92.4	92.3	0.37	0.076	0.118
Neutral detergent fiber ^8^	42.9	43.0	46.1	45.8	0.65	0.036	0.889
Non-fibrous carbohydrates	96.2	94.3	95.6	95.1	0.58	0.707	0.545
Total digestible nutrients	73.9	73.7	74.1	72.5	0.45	0.320	0.431

^1^ SEM, standard error of the mean; regression equations: ^2^ EE intake = 67.12 − 0.0029X, R^2^ = 0.79; ^3^ NDF intake = 594.51 + 0.7218X, R^2^ = 0.98; ^4^ NDFi intake = 245.05 + 0.2763X, R^2^ = 0.97; ^5^ NDFpd intake = 349.46 + 0.4455X, R^2^ = 0.98; ^6^ NFC intake = 645.27 − 0.6923X, R^2^ = 0.97; ^7^ DM digestibility = 68.65 − 0.1130X, R^2^ = 0.82; ^8^ NDF digestibility = 42.70 + 0.0177X, R^2^ = 0.97.

**Table 3:** Feeding behavior of lactating goats fed diets with increasing levels of licury cake.

Variable	Licury Cake (g kg^−1^)				SEM ^1^	*p*-Value	
	0	67	133	200		Linear	Quadratic
	Time per activity (min day^−1^)						
Feeding	292.1	297.1	296.3	300.4	8.10	0.669	0.974
Rumination	462.5	476.7	439.6	432.1	10.78	0.084	0.565
Idling	685.0	666.3	704.2	707.5	13.13	0.228	0.569
	Feeding efficiency (g h^−1^)						
Dry matter	340.8	340.7	340.9	338.9	14.50	0.944	0.952
Neutral detergent fiber ^2^	125.5	134.8	146.5	155.8	6.48	0.005	0.999
	Rumination efficiency (g h^−1^)						
Dry matter	211.9	210.5	219.9	224.6	6.42	0.258	0.742
Neutral detergent fiber ^3^	77.9	83.4	94.5	103.2	3.01	<0.001	0.665
	Periods per activity (N° of episodes day^−1^)						
Feeding	17.79	15.42	15.42	15.17	0.69	0.053	0.228
Rumination	26.50	28.00	26.92	27.83	0.81	0.559	0.793
Idling	35.33	35.83	34.08	35.58	0.77	0.850	0.672

^1^ SEM, standard error of the mean; regression equations: ^2^ NDF feeding efficiency = 125.28 + 0.1540X, R^2^ = 0.99; ^3^ NDF rumination efficiency = 76.69 + 0.1305X, R^2^ = 0.98.

**Table 4:** Milk composition and production of lactating goats fed diets with increasing levels of licury cake.

Variable	Licury Cake (g kg^−1^)				SEM ^1^	*p*-Value	
	0	67	133	200		Linear	Quadratic
4% Fat-corrected milk yield (g day^−1^)	1032.4	1013.5	1041.2	982.2	70.94	0.664	0.579
	Milk composition (%)						
Fat	3.9	4.5	4.2	4.2	0.12	0.486	0.102
Protein	3.5	3.5	3.5	3.4	0.06	0.151	0.807
Lactose	4.5	4.4	4.5	4.5	0.02	0.882	0.937
Total solids	12.6	13.1	13.0	12.8	0.17	0.690	0.088
Total solids non-fat	8.7	8.7	8.7	8.6	0.06	0.129	0.505
Urea (mg dL^−1^) ^2^	26.9	28.9	29.4	29.5	0.45	0.026	0.881
Somatic cell count (×1000 mL^−1^)	1319.3	708.2	576.8	1400.8	140.49	0.952	0.093
Somatic cell score	6.72	5.82	5.53	6.81	0.56	0.345	0.162

^1^ SEM, standard error of the mean; regression equations: ^2^ ureic nitrogen = 27.24 + 0.140X, R^2^ = 0.99.

**Table 5:** Blood metabolites of lactating goats fed diets with increasing levels of licury cake.

Variable	Licury Cake (g kg^−1^)				SEM ^1^	*p*-Value	
	0	67	133	200		Linear	Quadratic
Albumin (g dL^−1^)	1.5	1.4	1.6	1.5	0.09	0.348	0.411
Globulin (g dL^−1^)	4.4	4.2	4.7	4.6	0.19	0.935	0.939
Albumin:globulin ratio	0.3	0.4	0.3	0.3	0.06	0.884	0.756
Total proteins (g dL^−1^)	5.9	6.1	6.3	6.1	0.17	0.634	0.528
Urea (mg dL^−1^) ^2^	63.3	76.6	72.5	77.0	3.57	0.899	0.044

^1^ SEM, standard error of the mean; regression equations: ^2^ Urea = 64.52 + 0.1531X − 0.00049X^2^, R^2^ = 0.76.

**Table 6:** Nitrogen balance in lactating goats fed diets with increasing levels of licury cake.

Variable	Licury Cake (g kg^−1^)				SEM ^1^	*p*-Value	
	0	67	133	200		Linear	Quadratic
	Nitrogen (g day^−1^)						
Ingested	47.6	49.2	46.2	46.7	1.47	0.392	0.714
Excreted in feces	7.2	6.1	7.3	5.9	0.24	0.052	0.641
Excreted in milk	5.4	5.2	5.4	5.1	0.39	0.509	0.772
Excreted in urine	15.8	11.4	15.6	18.3	1.18	0.166	0.066
Retained	19.2	26.5	17.9	17.4	1.73	0.187	0.178
Digested	40.4	43.1	38.9	40.8	1.34	0.647	0.775

^1^ SEM, standard error of the mean.

## Data Availability

Not applicable.
